# Infant Distress in a Food Delay Task Changes With Development and Predicts Amount Consumed

**DOI:** 10.3389/fnut.2022.786022

**Published:** 2022-04-07

**Authors:** Sara F. Stein, Hurley O. Riley, Niko Kaciroti, Katherine L. Rosenblum, Julie M. Sturza, Ashley N. Gearhardt, Andrew C. Grogan-Kaylor, Julie C. Lumeng, Alison L. Miller

**Affiliations:** ^1^Department of Health Behavior and Health Education, University of Michigan School of Public Health, Ann Arbor, MI, United States; ^2^School of Social Work, University of Michigan, Ann Arbor, MI, United States; ^3^Department of Biostatistics, University of Michigan School of Public Health, Ann Arbor, MI, United States; ^4^Department of Psychiatry, Michigan Medicine, Ann Arbor, MI, United States; ^5^Department of Pediatrics, Michigan Medicine, Ann Arbor, MI, United States; ^6^Department of Psychology, University of Michigan College of Literature, Science, and the Arts, Ann Arbor, MI, United States

**Keywords:** delayed gratification, appetite, distress, infants, ATDG

## Abstract

**Objective:**

Eating behavior regulation emerges during early development and involves general self-regulation (emotional, behavioral), appetite regulation (homeostatic metabolic need) and appetite self-regulation (including both Bottom-Up Food Approach and Bottom-Up Food Avoidance and top-down purposeful self-control of eating). Limited research has investigated developmental trajectories of the regulation of eating behavior before the preschool years. The current study used a novel food delay task to assess infant distress as an early emerging marker of eating behavior regulation constructs across early infancy and examine associations with amount of milk consumed.

**Method:**

Mother-infant dyads (*n* = 179) completed the Ability to Delay Gratification for Food in Infants Task (ATDG-FIT) at 2 weeks, 8 weeks, and 16 weeks of age. The ATDG-FIT required infants to wait before being fed while their bottle was present, but not accessible (3-min Pre-Feeding Delay). After this, the infant was fed for 1 min, then the feeding was paused for 30 s (Mid-Feeding Delay). Infant distress was coded during each feeding delay period and the amount of milk consumed was measured.

**Results:**

The mean proportion of distress during the Pre-Feeding Delay period decreased from 8 to 16 weeks of age (*F*(2,230) = 15.02, *p* < 0.001), whereas the mean proportion of distress during the Mid-Feeding Delay increased from 2 to 8 weeks of age (*F*(2,230) = 27.04, *p* < 0.001). There was a positive interaction between distress during Mid-Feeding Delay and infant age predicting the amount consumed in the protocol (ß = 0.30, *p* = 0.022), suggesting that the association between distress during this part of the task and amount consumed strengthens as infants get older.

**Conclusion:**

The ATDG-FIT may be an effective method to assess emerging eating behavior regulation constructs during early infancy.

## Introduction

Obesity established during early childhood is often sustained (1), and difficulties with regulation of eating behavior are hypothesized to promote rapid weight gain (2). For example, it has been proposed that individuals who are more successful at delaying gratification are better able to resist eating tempting foods, thus reducing their risk of overweight and obesity over time (3). The ability to delay gratification (ATDG), defined as the ability to postpone immediately available rewards in order to attain a desired outcome (4), has thus been studied extensively among preschool-aged children, most often using food stimuli (3, 5, 6). A longitudinal study of 805 children found that those who demonstrated poorer ATDG for food at 4 years of age were more likely to be overweight at 11 years of age (6), and a separate longitudinal study of 164 children found that poorer ATDG for food at 4 years of age was associated with higher BMI 30 years later (3). Limited research has investigated ATDG prior to age 3 years (7). Of studies that have done so, poorer ATDG (for a gift, not food) at age 2 years was associated with higher BMI at age 5 years (8) and age 10 years (9). Another study found that greater ATDG for food at age 2 years was associated with lower concurrent BMIz (10). Yet, very little is known about early precursors of ATDG for food or how younger children and infants may respond to ATDG-for-food tasks.

The regulation of eating behavior involves a nuanced interplay between homeostatic, hedonic, and cognitive control factors, and is not well-characterized prior to age 12 months (11, 12). Yet, infancy is an important developmental period during which to identify indicators of emergent eating behavior regulation, as it is a time of rapid development and changes in eating and growth. Behaviors that can be measured during infancy may be relevant for understanding, predicting, and ultimately shaping later eating behavior regulation. For example, Neale et al. (13) used a spoon grasping task with 12-month-olds which required inhibiting their response to grasp a spoon facing the wrong direction, and instead grasp the spoon handle in order to obtain food. Responses on this task at 12 months predicted ATDG for food at 24 months. Assessing infant responses to tasks that are designed to elicit early indicators of eating behavior regulation may inform efforts to identify early emerging individual differences that may signal later poor eating behavior regulation and possible risk for obesity.

In their recent overviews of the literature, Russell and Russell (2, 11) provided an overarching framework for considering developmental changes in capacity to regulate one’s eating that included both general self-regulation (e.g., cognitive control) and self-regulation of appetite specifically (including homeostatic need and hedonic factors). They also noted a lack of consistency in the terms used to describe key constructs in the field. We use the term “eating behavior regulation” to capture the conceptual domain of eating regulatory capacity and expand on their framework by considering how relevant constructs emerge early in development and whether they may be assessed in ATDG-for-food task (see Table 1). The framework distinguishes General Self-Regulation (GSR), which includes regulation of emotions, cognition, and behavior (for infants, these are co-regulated with a caregiver), from Appetite Regulation (AR), which includes metabolic or homeostatic needs such as hunger and thirst, and Appetite Self-Regulation (ASR), which is comprised of the factors of appetitive traits characterized by propensity for food reward or “Bottom-Up Food Approach” and avoidance of food or “Bottom-Up Food Avoidance” and Top-Down regulation (i.e., goal-directed behavior to inhibit food intake). Traditional ATDG-for-food tasks (2) are thought to potentially measure GSR, Bottom-Up Food Approach, Bottom-Up Food Avoidance, and Top-Down ASR, but are not thought to measure AR. Although young infants do not yet have the developmental capacity for intentional choice to delay gratification (Top-Down ASR; 14), AR is highly significant in young infants for whom homeostatic need is a critical driver of eating behavior (15). Therefore, using ATDG-for-food tasks to assess eating behavior regulation in infants must consider not only the task stimuli (i.e., food vs. non-food) but also relevant infant developmental capacities.

**TABLE 1:** Constructs relevant to emerging eating behavior regulation; adapted from Russell and Russell.

Construct	Definition and examples [from Russell and Russell (2, 11)]	Could ATDG-FIT index?
General Self Regulation (GSR)	Capacity to self-regulate emotions, cognition, and behavior in relation to food (or non-food) stimuli (e.g., executive functioning; emotion regulation). Includes early infant self-soothing capacity and co-regulation with parent.	Yes
Appetite Regulation (AR; Russell and Russell)	Homeostatic need (e.g., long-term energy reserves, nutrient sensing and availability, metabolic requirements; short- and long-term energy homeostasis; hunger)	Yes
Bottom-Up Appetite Self Regulation (ASR) – Food Approach	Appetitive traits characterized by food-approach (e.g., food responsiveness, reward sensitivity, enjoyment)	Yes
Bottom-Up Appetite Self Regulation (ASR) – Food Avoidance	Appetitive traits characterized by food-avoidance (e.g., picky eating, food fussiness, slowness in eating)	No
Top-Down Appetite Self Regulation (ASR)	Purposeful inhibitory control of food intake (i.e., cognitive control of food intake for purposes of health, weight control; intentional choice; goal directed)	No

The developmental capabilities of infants younger than 6 months require that responses to ATDG tasks are captured through infant negative affect or frustration, as such tasks involve tolerating the typically unpleasant state of waiting for a desired outcome (16, 17). Regulation of negative affect in infancy, in coordination with a caregiver, is an important indicator of emerging GSR (18). For instance, a longitudinal study from ages 18 to 48 months found that a shorter duration of anger during a gift delay task was associated with a longer duration of distraction, a GSR strategy, at all timepoints (19). Distraction may aid in ATDG by allowing children to strategically shift their attention away from the desired object and reduce expression of negative affect associated with the frustrating situation. In contrast, toddlers who are unable to shift their attention away from the immediate desire are less able to tolerate the frustrating situation without negative affect (19, 20). As affect and attention regulation are closely connected during infancy (21), investigating infant distress during a delay task can give insight into an infant’s ability to cope with a frustrating situation and capacity for ATDG. With regard to implications for eating and appetite, although researchers have found that infants who are reported by parents to display distress in response to (non-food) limitations gained weight faster (22) and gained more body fat (23), no research has considered infants’ observed negative affect during a food delay task.

Considering responses to an ATDG-for-food task in infants younger than age 6 months also requires consideration of the fact that while infant distress is a very early indicator and precursor of later GSR, it is also an essential indicator of hunger, an aspect of AR (15) and a fundamental element of the mammalian system that maintains energy homeostasis (2). Distress vocalizations that elicit caregiving and feeding behaviors are essential to survival and are therefore tightly regulated by physiologic indicators of caloric need (i.e., hunger and satiety). In human infants and animal models, a low blood glucose reliably initiates feeding, generally through the onset of crying (24), while cholecystokinin (CCK), released from the small intestine in response to milk feeding, suppresses feeding, reduces seeking of feeding-related stimuli, pacifies crying, and causes sedation (25–28). These biological pathways result in a tight integration of hunger, satiety, and distress (29, 30).

Finally, individual differences in Bottom-Up Food Approach and Bottom-Up Food Avoidance, or appetitive traits such as food reward and avoidance have been studied in older children and identified as promoting or reducing risk for obesity, respectively (31–33). Russell and Russell (11) suggest that in older children, Bottom-Up Food Approach and Bottom-Up Food Avoidance processes could drive some of the mixed findings observed between Top-Down ASR and weight outcomes. Although less research has been conducted in infants, there is emerging evidence that appetitive traits, or Bottom-Up Food Approach indicators (e.g., food responsiveness) are associated with eating in the absence of hunger (34) as well as weight (35) within the first year of life. It is therefore also important to consider how Bottom-Up Food Approach could be a driver of infant response to ATDG-for-food tasks during very early infancy.

### Current Study

The goal of the current study was therefore to examine infant distress in a novel ATDG-for-food task – the ATDG-FIT – as a marker of early eating behavior regulation, specifically GSR, AR, and Bottom-Up ASR. We developed the ATDG-FIT to parallel ATDG-for-food tasks in older children so we could determine how infant responses in the ATDG-FIT would evolve across the first year of life and lay the groundwork to test whether they would relate to later ATDG performance. As noted in Table 1, we anticipate that the ATDG-FIT in early infancy (prior to 6 months of age) may assess GSR, AR, and Bottom-Up food approach, but not Top-Down ASR, given infants’ limited cognitive capacity for choice to delay (14). We used a longitudinal design and assessed infant distress while waiting to consume milk under two conditions (Pre-Feeding and Mid-Feeding Delay) across three timepoints (infant age 2, 8, and 16 weeks). The ATDG-FIT included both Pre-Feeding and Mid-Feeding Delay on the premise that differences in energy homeostasis (i.e., AR) under these two conditions may account for possible differential associations between distress and milk intake. We hypothesized that infants would show less distress during the delay periods as they grew older, reflecting improving GSR, and that decreased distress during the Pre-Feeding Delay specifically could reflect reduced acuity of metabolic need (i.e., changes in AR). We did not have specific hypotheses about change in Bottom-Up Food Approach across development. We also examined whether infant distress during the delay periods predicted the amount of milk infants consumed in the protocol and whether this association changed across early development. Overall, we hypothesized that there would be positive associations between distress during either feeding delay and amount of milk consumed across all three ages, reflecting the possibilities that more limited GSR could drive increased consumption as a soothing mechanism, that greater metabolic need (AR) could drive increased consumption, and that higher Bottom-Up Food Approach could drive greater demand and increased consumption.

## Materials and Methods

### Recruitment and Participants

Mother-infant dyads were recruited from a community in the Midwest United States through flyers, postcards, and social media. Mothers provided written informed consent for themselves and their infants. The study was approved by the University of Michigan Medicine Institution Review Board (IRB MED). Inclusion criteria for the study consisted of the following: (1) Child was born at 37.0 – 42.0 weeks gestation with weight appropriate for gestational age, and no significant perinatal or neonatal complications. Participants were excluded from the study if they met any of the following criteria: (1) Mother is not fluent in English; (2) infant is not the biological child of the mother; (3) mother < 18 years old; (4) medical problems or diagnosis affecting current or future eating, growth, or development; (5) child protective services involvement in the neonatal period; and (6) infant does not consume at least two ounces in one feeding from an artificial nipple and bottle at least once per week. Dyads were recruited to begin the study when infants were 2 weeks of age. To facilitate recruitment, families could also enter the study at infant age 8 weeks (2 months) or 16 weeks (4 months). Data were collected for each family at the infant’s first assessment point (termed “baseline”) and as described at each timepoint thereafter. Infants who were breastfed at all three timepoints were dropped from study analyses because there were no available objective measurements of amount consumed. Infants excluded from analyses for this reason (*n* = 106) did not significantly differ from those included in the analyses on any of the study variables of interest (all *p*’*s* ≥ 0.05). Infants who were bottle-fed for at least one of the three timepoints were retained as the analytic sample for the current study (*n* = 179).

### Procedure

Research assistants visited families’ homes and mothers completed several questionnaire-based measures at baseline. Mothers and infants also completed the ATDG-FIT in their home at baseline and subsequent timepoints (i.e., 2-, 8-, and 16-weeks), which was video recorded for observational coding. The ATDG-FIT was conducted at the point during the home visit when mothers indicated that they thought the infant was hungry based on the infant fussing or crying, which have been identified as relatively reliable indicators of infant hunger in prior literature (15). The time of day at which the ATDG-FIT was also recorded.

#### Ability to Delay Gratification for Food in Infants Task

The goal of the ATDG-FIT was to assess infant behaviors when the infant is not allowed to eat immediately (see Table 2, for task elements and sequence). Mothers were instructed to prepare to feed the infant their usual milk (either breast milk or formula) and usual bottle, prepared as they usually would, or prepare to breastfeed. Research assistants instructed the mothers, “we will ask you to prepare a bottle for [infant’s name]. Once the bottle is prepared, we will ask to set the bottle on the table or in the baby’s view for the first few minutes of the protocol. We want to make sure that [infant’s name] can see the bottle but not reach it. If [infant’s name] become upset, you can use any non-feeding method you’d like to soothe them.” Mothers who chose to breastfeed were instructed to hold but not feed their infant for this segment. Mothers were also instructed that they could have a pacifier nearby, which they could use only during the pacifier period of the protocol, if desired. The research assistant stayed behind the video camera and did not engage with the dyad during the protocol. Research assistants asked the mother when their infant was hungry and the protocol began when the mother indicated that the baby was hungry. The mother then held their infant for 3 min while the bottle was present and visible to the infant but not reachable (Pre-Feeding Delay). If the mother elected to breastfeed, then the mother held their infant without feeding. After the 3-min Pre-Feeding Delay, if the mother had a pacifier, the research assistant told the mother, “you can offer [infant’s name] the pacifier now.” The mother was given 2 min to offer the pacifier or continue holding the baby. Mothers without a pacifier continued to hold their baby. After 2 min, the mother was instructed, “you can feed [infant’s name] now for 1 min.” The research assistant then timed the infant feeding for 1 min. After the 1-min feeding period, the mother was asked to stop feeding for 30 s (Mid-Feeding Delay) by giving the bottle to the research assistant or covering access to their breast. The research assistant then placed the bottle where it was visible to the infant but not reachable. After the 30-s Mid-Feeding Delay, the mother was asked to continue feeding as she typically would until the feeding was complete. For infants who were bottle-fed, the bottle was weighed before and after the protocol to measure the amount consumed by the infant during the protocol.

**TABLE 2:** Ability to delay gratification for food in infants task (ATDG-FIT).

Segment	Length of time
Pre-Feeding Delay (bottle present/visible, but no access)	3 min
Bottle visible, but no access, pacifier optional	2 min
Bottle given	1 min
Mid-Feeding Delay (bottle removed to pause feeding)	30 s
Feeding until completion	Until dyad completes feeding

*We analyzed infant distress during the “Pre-Feeding Delay” and “Mid-Feeding Delay” segments.*

### Measures

#### Distress

Infant distress, defined as displays of negative affect, was coded in 10-s intervals as none (0), mild (1), or moderate/intense (2) based on facial, vocal, and body movement indicators. Mild distress included instances of whimpering, mild fussing, and facial expressions or body movements indicating distress or frustration (e.g., downturned mouth, mild squirming). Moderate/intense displays of negative affect included stronger displays of distress such as hard crying, active squirming or arching back while crying, or screaming. In the current study we considered any distress as an indicator so we created a composite code to indicate distress present (1; mild or moderate/intense distress) or not present (0; no instances of distress). Undergraduate research assistants, who did not administer the protocol, were trained to achieve interrater reliability (κ > 0.70) on coding at each of the three timepoints prior to coding the videos. Approximately 20% of videos were double-coded throughout the coding process to assess interrater reliability at the 2-week (κ = 0.73), 8-week (κ = 0.75), and 16-week (κ = 0.78) timepoints. For data analysis purposes, we calculated the proportion of distress in each segment (i.e., the total number of intervals with distress present divided by the total number of coded intervals). We analyzed infant distress during the 3-min Pre-Feeding Delay (i.e., bottle visible, but no access) and 30-s Mid-Feeding Delay (i.e., bottle removed to pause feeding) periods, as these two segments required infants to wait without milk or a pacifier being offered. Hereafter, we refer to these variables as Pre-Feeding Delay Distress and Mid-Feeding Delay Distress.

#### Amount Consumed

Amount of breastmilk or formula consumed by the infant in grams was calculated by subtracting weight of the bottle and contents remaining from the initial weight of the bottle and contents. Bottles were weighted using a Taylor TE32FT digital scale (2lb × 0.01oz/1 kg × 0.5 g; accurate to ± 0.5 g). It was only possible to directly calculate the amount consumed variable in this manner for infants who were bottle-fed. Values for amount consumed were imputed for any timepoints when infants were breastfed, but this was true only for those infants who were bottle-fed for at least one of the three study timepoints.

#### Covariates

Infant weight-for-length z-score (WLZ) was included as a covariate, given associations with infant consumption amount (36). Infant weight and length were measured at all timepoints twice during the same visit and averaged; if the two measurements differed by >0.1 kg for weight or >0.2 cm for length, then a third measurement was obtained and averaged. Measurements were used to calculate WLZ based on the World Health Organization growth charts (37). Infant feeding mode (breast or bottle-fed), and milk type (breast milk or formula) used in the ATDG-FIT were also included as covariates. Infant feeding mode was coded as breastfed or bottle-fed (breastfed = 0; bottlefed = 1) at each timepoint. Infant milk type was coded as breast milk or formula (breast milk = 0; formula = 1) at each timepoint. Time elapsed since last feeding was also included as a covariate, given well-established associations between feeding intervals and amount consumed (38–40). Mothers reported the time of the infant’s last feeding prior to the start of the ATDG-FIT, from which time elapsed since last feeding was calculated.

#### Demographics

Mothers reported whether they were Hispanic or not, as well as their race, choosing from United States Census categories White, Black, American Indian or Alaska Native, Asian, Native Hawaiian or Pacific Islander, Multiracial (and if so, which races), and Other; categories were collapsed to indicate race/ethnicity (White non-Hispanic, Black non-Hispanic, Hispanic any race, and other non-Hispanic [American Indian/Alaskan Native, Asian, Native Hawaiian/Pacific Islander, Multiracial]. Mothers reported on family income, number of individuals living in the household, and infant sex at baseline. Income to needs ratio (ITNR) was calculated by dividing income by the poverty income threshold for a household of that size in the given year; an ITNR of 1.0 indicates that a household is living at the poverty level, with higher values indicating greater income-to-needs (i.e., lower poverty) (41).

### Analysis Plan

Descriptive statistics were used to characterize the sample and bivariate statistics were used to assess associations among key variables. To examine developmental change, analyses of variance (ANOVAs) using repeated measures were used to assess differences in Pre-Feeding Delay Distress and Mid-Feeding Delay Distress) across the 3 ages. Repeated measure ANOVAs were also used to assess differences in mean amount consumed across the 3 ages.

To examine whether Pre-Feeding Delay Distress or Mid-Feeding Delay Distress related to amount consumed and whether the association changed with age, multilevel modeling (MLM) in Stata 17 (42) was used to examine time-variant predictors (see below) of amount consumed over time while accounting for possible correlation between repeated measures (43). MLM analyses also accounted for the fact that the number of weeks differed between assessment timepoints by using infant’s exact age at the time of the assessment. Predictors of interest were infant age, Pre-Feeding Delay Distress, and Mid-Feeding Delay Distress. Covariates were time varying infant WLZ, feeding mode (breast- vs. bottlefed), milk type (breast milk vs. formula), and time elapsed since last feeding that occurred prior to the ATDG-FIT.

Multi-level model was used to examine predictors of the amount consumed across development (i.e., trajectory of amount consumed) for Pre-Feeding Delay Distress or Mid-Feeding Delay Distress. This MLM included an infant age by distress interaction term in order to test whether the association between Pre-Feeding Delay Distress or Mid-Feeding Delay Distress and amount consumed changed as infants grew older. A positive interaction between infant age and either distress variable would indicate that the strength of the association between distress and amount consumed increased as infants grew older. The model to predict amount consumed was estimated including time variant independent variables using the following equation:

*y*_*it*_ = β_0_ + β_1_(Infant age) + β_2_(Pre-Feeding Delay Distress) + β_3_(Mid-Feeding Delay Distress) + β_4_(Pre-Feeding Delay Distress*Infant age) + β_5_(Mid-Feeding Delay Distress*Infant age) + β_6_(Feeding mode) + β_7_(Milk type) + β_8_(WLZ) + β_9_(Time elapsed since last feeding) + *u*_0*i*_ + *e*_*it*_

### Missing Data Imputation

Missing values for amount consumed at the timepoints when infants were breastfed were handled alongside all other missing data using multiple imputation with covariates (44–46). Imputation was conducted based on the values of other independent variables included in the statistical model, following recommended methods (46). Amount consumed and milk type had between 20 and 72% missing data, with breastfeeding accounting for most missingness, as expected. Other variables had between 11 and 45% missing data. Twenty imputed datasets were created and then simultaneously analyzed in accordance with recommendations for the number of imputations (47). Regression coefficients and standard errors were averaged across regression models (44).

## Results

### Descriptive Statistics and Bivariate Associations

The total sample recruited consisted of 285 mother-infant dyads. Of these, 179 had data from at least one ATDG-FIT assessment at infant age 2 weeks (timepoint 1; *n* = 99), infant age 8 weeks (timepoint 2; *n* = 155), and/or infant age 16 weeks (timepoint 3; *n* = 157) and were included in the analytic sample for the current study (see Table 3). Of this sample, more than half of the infants were girls (53%) and 66% of mothers were White, non-Hispanic. The mean ITNR was 3.46 (*SD* = 2.23) for this sample, where values of 1.0 indicate that a household is living at the poverty level and higher values indicate greater income-to-needs (41). At 2 weeks, 41% of infants were bottle-fed during the ATDG-FIT; at 8 weeks, 52% were bottle-fed; and at 16 weeks, 64% were bottle-fed. The majority of dyads completed the ATDG-FIT protocol midday (*mean start time* = 12:46 PM, *SD* = 2 h and 20 min).

**TABLE 3:** Sample demographics and covariates of interest (*N* = 179).

Sample demographics collected at study entry for each family	*M*(SD)/%		
Infant sex (girls)	53%		
Mother race/ethnicity			
White, non-Hispanic	66%		
Black, non-Hispanic	17%		
Hispanic, any race	6%		
Other (American Indian/Alaskan Native, Asian, Native Hawaiian/Pacific Islander, Multiracial)	11%		
Income to needs ratio (ITNR)	3.46 (2.23)		

**Covariates of interest collected at each study wave**	**Timepoint 1 (2 Weeks)** ***n* = 99 *M*(*SD*)/%**	**Timepoint 2 (8 Weeks) *n* = 155 *M*(*SD*)/%**	**Timepoint 3 (16 Weeks) *n* = 157 *M*(*SD*)/%**

Infant age (weeks)	3.23 (0.98)	9.25 (1.47)	17.73 (1.83)
Infant weight to length z-score (WLZ)	−0.12 (1.07)	0.04 (1.03)	0.12 (0.96)
Feeding mode (Bottle)	41%^a^	52%^b^	64%^c^
Food type (Breast milk)	61%^a^	55%^a^	47%^b^
Pre-Feeding Delay distress (proportion of segment)	0.60 (0.34)^a^	0.58 (0.36)^a^	0.40 (0.38)^b^
Mid-Feeding Delay distress (proportion of segment)	0.32 (0.36)^a^	0.62 (0.40)^b^	0.60 (0.42)^b^
Amount consumed in grams	72.39 (38.31)^a^	85.12 (54.79)^b^	109.36 (62.91)^c^
Time elapsed since last feeding (minutes)	149.57 (63.49)^a^	164.50 (70.41)^ab^	172.29 (82.10)^b^

*Differing superscripts denote within-row significant differences. Different n’s denote the number of dyads with data for that study wave. Descriptives are presented on data prior to imputation. Income to needs ratio (ITNR) was calculated by dividing income by the poverty income threshold for a household of that size in the given year. ITNR is a commonly used metric to indicate the financial situation a family is in relative to needs. In terms of interpretation, an ITNR of 1.0 indicates a household is living at the poverty level; higher values indicate greater income (41).*

Bivariate analyses of the data prior to imputation indicated that covariates (feeding mode [breast- vs. bottlefed], and milk type [breast milk vs. formula] during the ATDG-FIT) were associated with key study variables of Pre-Feeding Delay Distress, Mid-Feeding Delay Distress, and/or amount consumed for at least one timepoint; for example amount consumed was positively associated with being bottlefed and consuming formula, and distress was positively associated with being bottlefed. Bivariate analyses also indicated that Pre-Feeding Delay Distress, Mid-Feeding Delay Distress, and amount consumed were all positively associated (all *p*’s reported < 0.05). Specifically, infant distress during Pre-Feeding Delay was positively associated with amount consumed at 16-weeks of age (*r* = 0.28, *p* < 0.001), and positively but not significantly associated with amount consumed at 2-weeks (*r* = 0.25, *p* = 0.09) or 8-weeks (*r* = 0.11, *p* = 0.26). Infant distress during Mid-Feeding Delay was positively and significantly associated with amount consumed at all timepoints (2-week *r* = 0.34, *p* = 0.02; 8-week *r* = 0.19, *p* = 0.04; 16-week *r* = 0.36, *p* < 0.001).

Means and standard deviations for all study variables at each measurement occasion prior to multiple imputation are summarized in Table 3. One-way repeated measures ANOVAs were conducted prior to multiple imputation to assess mean differences in key variables across timepoints (significant differences across timepoints noted in Table 3). Observed distress at both Pre-Feeding and Mid-Feeding Delay ranged from 0 to 1, reflecting large individual differences. Amount consumed differed across timepoints (*F* (2,130) = 22.31, *p* < 0.001). Infants consumed significantly more at 16 weeks compared to 8 weeks (*t* = 4.61, *p* < 0.001), at 16 weeks compared to 2 weeks (*t* = 6.25, *p* < 0.001), and at 8 weeks compared to 2 weeks (*t* = 2.87, *p* = 0.013). The percentage of infants who were breastfed and fed breastmilk declined across the three ages (see Table 3).

### Association of Pre-Feeding Delay Distress and Mid-Feeding Delay Distress With Age

**FIGURE 1:**
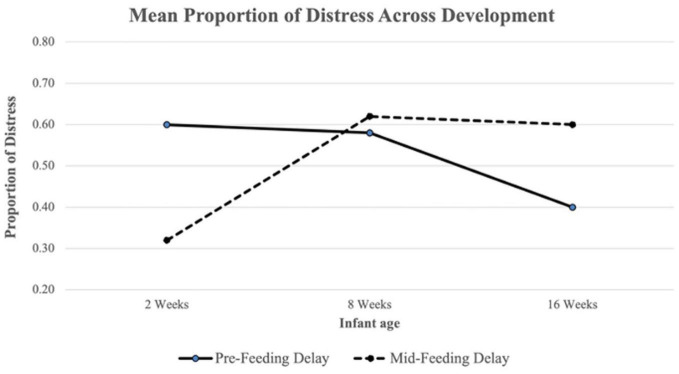
Mean proportion of Pre-Feeding and Mid-Feeding Delay distress across development. The proportion of distress in each delay segment was calculated as the total number of intervals with distress present divided by the total number of coded intervals.

ANOVA results revealed a difference in Pre-Feeding Delay Distress across timepoints (*F*(2,230) = 15.02, *p* < 0.001; see Figure 1 and Table 3). A Tukey *post-hoc* test revealed significantly lower Pre-Feeding Delay Distress at 16 weeks compared to 8 weeks (*t* = −4.79, *p* < 0.001) and at 16 weeks compared to 2 weeks (*t* = −4.47, *p* < 0.001). No significant difference was found between 8 and 2 weeks. There was also a difference in Mid-Feeding Delay Distress across ages (*F*(2,230) = 27.04, *p* < 0.001; see Figure 1 and Table 3). A Tukey *post-hoc* test revealed significantly higher Mid-Feeding Delay Distress at 16 weeks compared to 2 weeks (*t* = 6.21, *p* < 0.001) and 8 weeks compared to 2 weeks (*t* = 6.99, *p* < 0.001). No significant difference was found between 16 and 8 weeks.

### Association Between Distress and Amount Consumed Across Ages

**FIGURE 2:**
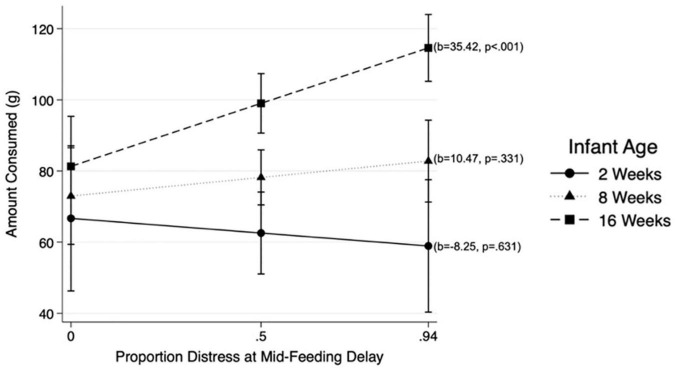
Associations between distress at Mid-Feeding Delay and amount consumed at different infant ages: 2, 8, and 16 weeks.

Results from MLM analysis (see Table 4) revealed a significant interaction between Mid-Feeding Delay Distress and infant age (ß = 0.298, *p* = 0.022, 95% CI = [0.45, 5.79]). The association between Mid-Feeding Delay Distress and amount consumed became stronger as infants grew older. Figure 2 illustrates that at infant age 2 weeks and 8 weeks, the amount consumed was not associated with Mid-Feeding Delay Distress, whereas at 16 weeks the amount consumed was positively associated with Mid-Feeding Delay Distress (see unstandardized beta values for each age in Figure 2). In this model, amount consumed was significantly associated with infant WLZ (ß = −0.156, *p* = 0.007, 95% CI = [−15.41, −2.48]), formula feeding during the protocol (ß = 0.228, *p* < 0.001, 95% CI = [12.45, 38.06), and time elapsed since last feeding (ß = 0.199, *p* < 0.001, 95% CI = [0.11, 0.26]), but not significantly associated with infant age, Pre-Feeding Delay Distress, age by Pre-Feeding Delay Distress interaction, Mid-Feeding Delay Distress, or feeding mode.

**TABLE 4:** Multilevel model estimating amount of milk consumed over time.

Fixed Effects	ß	SE	*t*	*P*-value	[95% CI]
Infant age	0.101	0.94	1.14	0.254	[−0.78, 2.93]
Pre-Feeding Delay distress	0.097	21.22	0.74	0.458	[−26.23, 57.84]
Infant age * Pre-Feeding Delay distress	0.005	1.50	−0.04	0.967	[−3.02, 2.90]
Mid-Feeding Delay distress	−0.101	19.51	−0.74	0.460	[−53.24, 24.27]
Infant age * Mid-Feeding Delay distress	0.298	1.35	2.31	0.022	[0.45, 5.79]
Infant weight for length	−0.156	3.29	−2.72	0.007	[−15.41, −2.48]
Milk type (breast milk = 0, formula = 1)	0.228	6.50	3.88	0.000	[12.45, 38.06]
Feeding mode (breast = 0, bottle = 1)	−0.064	9.73	−0.90	0.369	[−28.15, 10.58]
Time elapsed since last feeding	0.199	0.04	4.88	0.000	[0.11, 0.26]
Intercept	–	18.77	1.20	0.237	[−15.14, 60.05]

**Random effects**	**Estimate**		**SE**	**[95% CI]**	

Person level variance	27.38		4.11	[20.37, 36.79]	
Residual variance	40.76		2.66	[35.82, 46.39]	

*ß = Standardized Beta; SE = Standard Error; CI = Confidence Interval.*

## Discussion

The current study sought to investigate infant responses hypothesized to reflect emerging eating behavior regulation in a novel protocol – the ATDG-FIT. Specifically, we tested whether distress during a Pre-Feeding and a Mid-Feeding Delay changed with infant age, and whether distress would predict the amount of milk infants consumed during the protocol across early development. First, we found that infants showed distress in the ATDG-FIT as early as 2 weeks after birth. Second, we found that Pre-Feeding Delay distress decreased from 8 to 16 weeks, whereas Mid-Feeding Delay distress increased from 2 to 8 weeks of age. Third, the association between distress during Mid-Feeding Delay – but not Pre-Feeding Delay – and amount consumed became stronger with age such that it was present at 16 weeks, but not at 2 or 8 weeks. This association was present independent of infant WLZ, feeding mode, or milk type, and time elapsed since last feeding.

### Emergent Eating Behavior Regulation in the Ability to Delay Gratification for Food in Infants Task

Despite significant research on ATDG during early childhood, little work has examined responses to food delay tasks during infancy (48). Prior research has used familiar and motivating objects to test infant delay capacity (13), but researchers interested in the development of eating behavior regulation have emphasized the importance of using food-specific tasks (2, 10). The ATDG-FIT was therefore designed to mirror ATDG-for-food tasks in older children by using milk and a familiar object (bottle or breast) to elicit infant responses while waiting to be fed. We considered infant distress observed during specific sections of the protocol–Pre-Feeding Delay (i.e., when bottle was present, but not accessible) and Mid-Feeding Delay (i.e., when bottle was removed)–as indicating emergent eating behavior regulation, specifically GSR, AR, and Bottom-Up Food Approach. The infant distress observed during Pre-Feeding Delay may have indicated physiological hunger, or AR; indeed, distress is a critical infant hunger cue for caregivers during the earliest weeks of life that helps to ensure an infant will not starve (15, 24). In contrast, Mid-Feeding Delay captured infant behavior while waiting for food under different circumstances, after the infant has started feeding and may be less hungry (26, 49). Distress in this segment may have signaled greater frustration with the removal of the bottle and the need to wait for more milk, thus could be interpreted as implicating GSR rather than AR. Distress in either segment may also have indexed Bottom-Up Food Approach, or propensity for food reward. Thus, in addition to examining associations between ATDG-FIT responses and amount of milk consumed in the protocol as we did in the current study (see discussion below), it will be an important direction for future work to examine associations between individual differences in infant ATDG-FIT responses and other hypothesized early life indicators of eating behavior-related constructs, for example response to sweet taste (50) or sucking behavior in young infants (51), or reinforcing behavior paradigms in older infants (52, 53).

### Developmental Change in Responses to the Ability to Delay Gratification for Food in Infants Task

In terms of developmental change, consistent with our hypothesis, we found that distress during the Pre-Feeding Delay decreased as infants aged. There are several possible explanations for this observation, mostly related to changes in energy homeostasis with age (i.e., AR), suggesting infants may be more able to tolerate hunger while waiting for milk as they get older. Between ages 2 weeks and 3 months, infant adiposity nearly triples [from about 11 to 30% body fat; (54)]. Blood glucose correlates closely with adiposity (55), and declines in blood glucose initiate eating (24). Younger infants also have very high glucose turnover and less ability to mobilize hepatic stores efficiently (56). Due to this glucose physiology, younger infants experience a more acute and urgent need for feeding than do older infants. Declining Pre-Feeding Delay distress with age could also be explained by operant conditioning. CCK mediates learning of food cues in human infants and animal models (28, 49, 57) – calming in response to feeding cues is conditioned by the linking of the CCK-induced pacifying effects with feeding in very early infancy. The ability of the older, but not younger, infants to calm in response to milk cues may represent their greater exposure to and learning of food and feeding cues. Finally, this change could be due to the manner in which homeostatic states initiate feeding in older vs. younger infants. Specifically, in animal models, motivation to suck occurs from birth in response to presentation of a feeding cue, regardless of nutritional state; the association between motivation to suck and homeostatic caloric need only emerges in later infancy (58). Thus, while 2-week-old infants will invariably demonstrate distress (i.e., emitting vocalizations to cue feeding from the caregiver) simply at exposure to a feeding cue, older infants are more likely to demonstrate distress only when they are exposed to a feeding cue *and* have caloric need (26, 58, 59); isolating the roles of hunger, response to feeding cues, and how Bottom-Up Food Approach may relate to each of these processes would be interesting to examine further in future research.

In contrast to Pre-Feeding Delay, and somewhat counter to our hypothesis, distress during Mid-Feeding Delay significantly increased between 2 and 8 weeks of age. Potential explanations for this observation could relate to GSR, AR, and Bottom-Up Food Approach. First, this finding may in part reflect the development of object permanence throughout the first several months of infancy, a fundamental cognitive achievement that underlies later GSR capacities such as delay of gratification skills (60). In the current study, 2-week-old infants may have not been fully aware of the bottle continuing to exist after it was removed and, in turn, were less distressed than older infants after the bottle was removed. With ongoing cognitive development, however, it is possible that the infants became more aware of the bottle’s continued existence once it was removed, resulting in more Mid-Feeding Delay distress at older ages. Another explanation related to GSR could be due to changes in the nature of infant affect expression in response to violations of expectations across this developmental period. Anger and frustration can be elicited by goal blockage and are first detectable in response to goal blockage (e.g., arm restraint) by 2 months of age (61, 62). Goal blockage due to a feeding interruption may therefore simply not evoke this type of affective response until age 8 weeks, as infants may have limited awareness of their lack of control over the expected event (feeding) prior to this age (62). Thus, infants’ increasing cognitive and emotional capacity may result in greater, rather than lesser, distress in response to delay in this early period.

Additional explanations of the increase in Mid-Feeding Delay distress with age may involve AR. For example, as noted above, CCK is released in response to milk feeding, suppresses feeding, reduces seeking of feeding-related stimuli, pacifies crying, and causes sedation (25–28). Yet, CCK levels decline across infancy [CCK is 10 times higher in the newborn period than at age 9 months (63)] such that the sedating and pacifying effect of CCK released in response to milk feeding declines with infant age. Thus, while 1 min of milk ingestion at 2 weeks leads to immediate CCK release (28), with potent sedating and pacifying effects that maintain a calm behavioral state in the 2-week-old even during a 30-s feeding interruption, the CCK release in the 8-week-old and 16-week-old infant is less robust, and milk ingestion is not associated with potent calming effects during a mid-feeding interruption. Therefore, compared to the 2-week-olds, older infants may not have been as physiologically soothed by their milk intake during the Mid-Feeding Delay. In animal models, distress occurs when a schedule of positive reinforcement is interrupted (64). When expected delivery of food to a hungry animal is intermittent or interrupted, an increased level of activation is produced, which is channeled to other responses (65). When an animal is engaged in ingestive behavior under high-drive conditions (e.g., hunger), the animal’s high drive becomes directed to a displacement activity (64) – perhaps in this case, crying. It is possible that 2-week-old infants have not been exposed to the positive reinforcement schedule of feeding for enough duration or consistency to sufficiently evoke a response when that reinforcement schedule does not occur as anticipated; in contrast, by 8 weeks, the infant may have become accustomed enough to the positive reinforcement schedule of feeding that when it is unexpectedly interrupted, the infant becomes distressed.

Finally, it is also possible that distress during Mid-Feeding Delay may reflect Bottom-Up Food Approach factors such as food responsiveness (66). Milk feeding releases opioids (28), and the nature of opioid-mediated responses to sweet taste evolve across infancy (50). Infants have also been shown to “work” for milk by continuing to suck from a nipple with a smaller aperture as young as age 2 months (51), and by pressing a computer mouse button repeatedly as young as age 9 months (35). The greater infant distress exhibited at 8 and 16 weeks, compared to 2 weeks, may therefore reflect emergent reward sensitivity to food in early infancy, an aspect of Bottom-Up Food Approach.

### Distress and Milk Consumption in the Ability to Delay Gratification for Food in Infants Task

Regarding associations between ATDG-FIT distress displays and amount consumed, we found that distress during Mid-Feeding Delay associated with amount consumed only as infants grew older. Potential explanations for this observation may also reflect GSR, AR, and Bottom-Up Food Approach. Distress during Mid-Feeding Delay could indicate infants who are less able to tolerate delayed gratification (i.e., with poorer GSR) and who may become more likely to consume excess calories in response to frustration. Becoming reliant on food to soothe could establish links between behavioral distress and amount consumed and disrupt infant recognition of their own AR needs. Over time, this could translate to emotionally driven eating behaviors, such as eating in response to stress rather than hunger cues, and disrupted eating later in development (2). Infant negativity may result in parents’ overfeeding in attempts to soothe and quiet the infant (67, 68) resulting in heavier infant weight, especially for infants who are high in temperamental negativity (69). Although we found minimal associations with WLZ in the current study, such behaviors could promote excessive weight gain over time (2).

AR-related explanations for this association may also operate through a few different pathways. In animal models, although feeding releases CCK from early infancy, CCK causes calming and reduces the incentive salience for food in the earliest days of infancy, but does not affect volume intake; volume intake is only affected by CCK later in early infancy (26, 28). The dissociation of CCK with amount consumed in very early infancy is theorized to be due to either an unknown physiologic mechanism or that associative learning has not yet occurred (26). Second, in animal models, eating in the earliest stage of infancy is opportunistic – sucking occurs in response to opportunity; only later in infancy does the feeding system emerge as specifically regulated by peripheral feedback (49, 58, 59). Thus, the emergence of a linkage between distress and amount consumed in later infancy, but not early infancy, may reflect maturation of this system.

The association between Mid-Feeding Delay distress and amount consumed may also reflect changes in the reward value of food with development, an aspect of Bottom-Up Food Approach. Animal models have demonstrated that the components for reward-driven eating can change over time (70), reflecting sensitization of the dopamine system in response to repeated consumption of pleasurable foods. As the system becomes more sensitized, motivational drive (i.e., “wanting”) for food increases and can drive greater intake (70). The administration of smaller “priming” doses of a reward can further amplify motivational drive for more of the rewarding substance (71). Thus, the milk initially consumed prior to the Mid-Feeding Delay may prime the “wanting” system for more milk, increase distress, and drive greater intake when milk again becomes available. With development the reward system may become sensitized and this behavior may therefore become more evident in older infants who have had more opportunities to repeatedly experience food reward.

### Strengths and Limitations

The current study used a novel protocol and a longitudinal design to examine distress in response to a food delay and at an earlier age than has previously been considered. Such contextually specific, observational work is essential for understanding emerging eating behavior regulation and how GSR, AR, and Bottom-Up Approach may interact and underlie early individual differences in this domain. Prior research in this area has heavily relied on parent-reports, for example of general infant temperament [e.g., (22, 23)]. In contrast, the ATDG-FIT allowed us to objectively assess infant distress in a food-specific delay. Our finding of increased associations between amount consumed during the ATDG-FIT and distress during the Mid-Feeding, but not the Pre-Feeding Delay segment at older ages suggests that distress during each segment may reflect different aspects of emerging eating behavior regulation capacity. It will be important to track these behaviors over the first year of infancy in order to determine, for example, whether infant ATDG-FIT responses predict later ATDG for food. Further research is also needed to understand the integration of the affect regulation, nutritional homeostasis, and food reward systems in early infancy. As described, explanations for the observed phenomena include the development of cognitive capacity and affective and behavioral self-regulation (GSR), nutritional homeostatic controls (AR), and food reward sensitivity (Bottom-Up Food Approach) across early infancy. The manner in which these systems interact and potentially influence the development of one another is an important area for future work.

Despite the aforementioned strengths, there were several study limitations. It is possible that distress observed during the ATDG-FIT relates to dyadic processes that also shape infant response to food delays. Mothers were allowed to use any non-feeding/non-pacifier method to soothe the infant, hence the protocol likely captured variation in the mother’s ability to soothe their child without feeding or pacifier use. Given the resources required to conduct observational coding, we were unable to assess potentially confounding variables such as maternal attempts to soothe infant distress. Mothers were also instructed to complete the ATDG-FIT when they perceived their infants to be hungry, which could lead to bias, but we elected to do so to preserve ecological validity and based on findings that mothers are reasonably able to identify hunger (at least as compared to satiety (15). Furthermore, although distress during Mid-Feeding Delay became a stronger predictor of amount consumed in the protocol as infants aged, we did not objectively measure how it related to infant consumption outside of the protocol. We objectively measured amount consumed and parents were instructed to feed the infants as long as they deemed necessary, but we did not have data on whether the infants finished the bottle, so it is possible that the amount consumed variable could have been truncated and some infants may have even consumed more, if offered. Finally, although our multiple imputation approach was a strength, it is important to note that there was a substantial amount of missing data on the outcome variable (amount consumed), as it was not possible to measure amount consumed from infants who breastfed during the ATDG-FIT. Our exclusion criterion that the infant had not yet taken a feeding from an artificial nipple also may not generalize to all infants, although the majority of United States mothers who breastfeed expressed milk and feed their infant from a bottle at some point during early infancy (72).

## Conclusion

This study sought to investigate early emerging eating behavior regulation by examining distress during a novel food delay protocol, the ATDG-FIT, and whether distress predicted the amount of milk infants consumed in the protocol across early development. Findings highlighted the unique roles of both context and development, identifying different patterns of distress in the ATDG-FIT across time. Of note, the positive association between distress during Mid-Feeding Delay and amount consumed strengthened as infants grew older, suggesting that infant distress in this food delay context, even within the first 2 months of life, could signal possible risk for excessive consumption across early development. Our findings suggest that observing infant distress during the ATDG-FIT may be an important context in which to assess the interplay of infant GSR, AR and Bottom-Up Food Approach in order to characterize emerging regulation of eating behavior during early infancy.

## Data Availability Statement

The raw data supporting the conclusions of this article will be made available by the authors, without undue reservation.

## Ethics Statement

The studies involving human participants were reviewed and approved by the University of Michigan Medicine Institutional Review Board (IRB MED). Written informed consent to participate in this study was provided by the participants’ legal guardian/next of kin.

## Author Contributions

SS: contributed to drafting the initial manuscript, conducted the statistical analyses, and approved the final manuscript. HR: contributed to drafting the initial manuscript and approved the final manuscript. NK, JS, and AG-K: reviewed the statistical analyses and approved the final manuscript as submitted. KR and JL: conceptualized and designed the study, provided critical review of the manuscript, and approved the final manuscript. AG: provided critical review of the manuscript and approved the final manuscript. AM: conceptualized and designed the study, contributed to drafting the initial manuscript, provided critical review of the manuscript, and approved the final manuscript. All authors contributed to the article and approved the submitted version.

## Conflict of Interest

The authors declare that the research was conducted in the absence of any commercial or financial relationships that could be construed as a potential conflict of interest.

## Publisher’s Note

All claims expressed in this article are solely those of the authors and do not necessarily represent those of their affiliated organizations, or those of the publisher, the editors and the reviewers. Any product that may be evaluated in this article, or claim that may be made by its manufacturer, is not guaranteed or endorsed by the publisher.

## Abbreviations

ATDG: ability to delay gratification
CCK: cholecystokinin
ITNR: income-to-needs ratio
WLZ: weight-for-length *z*-score
MLM: multi-level model.
